# The Impact of the Natural Level of Blood Biochemicals on Electroencephalographic Markers in Healthy People

**DOI:** 10.3390/s24237438

**Published:** 2024-11-21

**Authors:** Laura Päeske, Hiie Hinrikus, Jaanus Lass, Toomas Põld, Maie Bachmann

**Affiliations:** 1 Department of Health Technologies, Tallinn University of Technology, 19086 Tallinn, Estonia; laura.paeske@taltech.ee (L.P.); jaanus.lass@taltech.ee (J.L.); maie.bachmann@taltech.ee (M.B.); 2 Meliva Medical Center, 10143 Tallinn, Estonia; toomas.pold@meliva.ee

**Keywords:** EEG processing, nonlinear marker, Higuchi’s fractal dimension, EEG complexity, blood biomarkers

## Abstract

This study aims to investigate the association between the natural level of blood biomarkers and electroencephalographic (EEG) markers. Resting EEG theta, alpha (ABP), beta, and gamma frequency band powers were selected as linear EEG markers indicating the level of EEG power, and Higuchi’s fractal dimension (HFD) as a nonlinear EEG complexity marker reflecting brain temporal dynamics. The impact of seven different blood biomarkers, i.e., glucose, protein, lipoprotein, HDL, LDL, C-reactive protein, and cystatin C, was investigated. The study was performed on a group of 52 healthy participants. The results of the current study show that one linear EEG marker, ABP, is correlated with protein. The nonlinear EEG marker (HFD) is correlated with protein, lipoprotein, C-reactive protein, and cystatin C. A positive correlation with linear EEG power markers and a negative correlation with the nonlinear complexity marker dominate in all brain areas. The results demonstrate that EEG complexity is more sensitive to the natural level of blood biomarkers than the level of EEG power. The reported novel findings demonstrate that the EEG markers of healthy people are influenced by the natural levels of their blood biomarkers related to their everyday dietary habits. This knowledge is useful in the interpretation of EEG signals and contributes to obtaining information about people quality of life and well-being.

## 1. Introduction

Electroencephalographic (EEG) signals reflect the functional state of the brain. Electroencephalography, a non-invasive and inexpensive method, has been widely used for diagnostics in clinical neurophysiology [1]. During the last decades, advanced methods in EEG processing have made it possible to apply electroencephalography for various purposes, from the detection of mild alterations in EEG signals in mental disorders to the use of EEG signals in brain–computer interfaces and other devices [2,3,4,5].

### 1.1. The Impact of External Factors on EEG Markers

The long-term reliability of EEG markers is supported by the studies of several authors [6,7,8,9,10]. However, EEG markers depend on some factors other than brain health. As the majority of physiological markers, EEG markers depend on age and gender [11,12,13,14,15]. EEG markers are also affected by the intake of alcohol, coffee, and drugs [16,17,18,19]. A recent publication demonstrates that dietary choices affect biological processes related to mental health and cognition [20].

EEG markers reflect the functioning of the brain and enable the evaluation of the state of the brain in metabolic encephalopathies related to an imbalance of chemicals in the blood [21]. Different blood biochemical markers have been demonstrated to be related to specific neurological diseases.

There is a possibility that the EEG markers of healthy people can depend on the level of different blood biochemicals related to their dietary habits. There is limited knowledge about the impact of blood biochemical markers on healthy brain functioning and human well-being.

### 1.2. The Impact of Blood Biomarkers on EEG Markers

The functioning of the brain is supported by the continuous absorption of necessary chemicals from the blood. Several molecules in the blood are important for the normal functioning of the brain, and biochemical imbalances can lead to neurological or mental disorders.

Blood glucose (Gluc) is the main source of energy for the brain. Abnormally low blood glucose levels, or hypoglycemia, cause serious disorders in brain functioning and alterations in EEG signals [22]. The impact of hypoglycemia on EEG markers has been investigated for decades [23,24,25,26,27,28,29]. Berger first observed slow brain functioning induced by hypoglycemia in 1937 [23]. A significant increase in low alpha (8–10 Hz) and theta (4–8 Hz) power in the whole brain was observed after the consumption of a glucose-rich drink following a fasting period of at least 8 h [26]. EEG power in all frequency bands has been reported to be related to glucose fluctuations in young adults with type 1 diabetes during sleep [27]. An experimentally elevated blood glucose concentration in the blood caused alterations in the alpha oscillations and the aperiodic signal components of resting-state EEG activity in healthy adults [28]. Experimental results demonstrated that the threshold of blood glucose concentration causing EEG changes during hypoglycemia varies between individuals but does not depend on age, duration of diabetes, insulin dose, hemoglobin concentration, initial blood glucose concentration, rate of fall of blood glucose concentration, or appearance of symptoms and signs of hypoglycemia [29].

Blood serum protein (Prot) is a complex of prealbumin, albumin, and globulins. Protein plays an important role in brain metabolism, supporting cognitive function and mental health [30]. Early experiments in rats demonstrated that protein malnutrition resulted in increased theta functioning (5–8 Hz) in the hippocampus [31]. A longitudinal 49-year lifelong study of resting-state EEG activity in 66 individuals with protein malnutrition limited to the first year of life showed abnormalities in childhood, including a developmental delay in alpha rhythm maturation and an insufficient decrease in beta functioning that may be correlated with cognitive decline [32].

The brain contains the highest level of lipoproteins in the body of humans. Lipoproteins (Lp) are complex structures composed of apolipoproteins and lipids. According to their size, density, and composition, lipoproteins in the blood are divided into four main classes: chylomicrons, very low density lipoprotein (VLDL), low-density lipoprotein (LDL), and high-density lipoprotein (HDL) [33]. Elevated levels of lipoprotein and abnormal cholesterol metabolism can result in serious neurodegenerative disorders [34,35]. A high level of cholesterol is excitotoxic for neuronal cells and can be related to epileptic seizures [36,37]. Only a few data are available about the relation between the level of cholesterol and EEG activity. Increased cholesterol has been reported to accelerate EEG oscillations from 7–10 Hz (theta) to 14–21 Hz (beta) in rapid eye movement sleep [37].

C-reactive protein (CRP) is a protein produced by the liver. CRP is in clinical use as a biomarker of inflammation in the body, and the level of CRP increases with inflammation [38]. The CRP levels in blood increase in epileptic patients compared to healthy controls [39]. Experimental results demonstrated that CRP concentration and EEG activity are affected in epilepsy [40]. Despite information about a direct relationship between CRP and EEG being missing, the existing results on the changes of CRP levels in epilepsy suggest that EEG markers could be affected by CRP concentration.

Cystatin C (CysC) is a protein produced by body cells. The kidneys regulate the level of cystatin C in the blood [41]. Cystatin C is implicated in neurodegenerative processes such as Alzheimer’s disease and dementia in aging [42,43]. The level of cystatin C is associated with the risk of mild cognitive impairment [44]. The involvement of CysC in brain neuronal functioning allows us to suggest that CysC could be related to EEG markers.

A review of the literature did not identify EEG studies on the natural level of blood biomarkers. No information is available about the association between the natural level of blood biomarkers and EEG activity in healthy people.

### 1.3. The Rationale for Investigating Blood Biomarkers in Healthy People

The majority of the performed studies were aimed at investigating the impact of blood biomarkers on various neurological diseases. The relationship between biomarkers and EEG activity was not always specifically investigated in these studies. The studies on the relationship between EEG signals and glucose or protein in healthy people used the experimental alteration of biomarker levels [26,28,31]. All previous studies investigated the impact of one biomarker, and their results are hardly comparable due to the very different methods used.

In previous studies, only the impact of blood biomarkers on the level of EEG band powers was reported. The existing knowledge about the relationship between blood biomarkers and EEG activity is limited to a linear approach describing EEG activity in the frequency domain using EEG frequency band powers as markers. No information is available about the relationship between blood biochemistry and the temporal dynamics of EEG describing different aspects of brain functioning compared to frequency band powers. To the best of our knowledge, nonlinear EEG complexity markers have not been used to investigate the impact of blood biomarkers on EEG markers.

This study aims to investigate the impact of the natural level of blood biomarkers on EEG markers in healthy people. Seven different blood biomarkers, i.e., glucose, protein, lipoprotein, HDL, LDL, C-reactive protein, and cystatin C were selected and analyzed using an identical experimental protocol and signal processing methods. Parallel to EEG frequency band powers, Higuchi’s fractal dimension, a most relevant nonlinear dynamics marker describing the complexity of EEG activity, was selected to reflect brain functioning in the current study [45].

## 2. Materials and Methods

### 2.1. Participants

The study was performed on healthy volunteers. The selected participants declared no previous neurological or psychiatric diseases, brain injuries, use of narcotics or psychotropic medications, diabetes, and chronic cardiovascular, kidney, or liver disease. In total, 52 participants were selected for the study, 34 males and 18 females. The average age of the participants was 47.7 ± 11.4 years. Before the measurements were performed, all participants read the information sheet describing the aim and the protocol of the study and signed a written consent agreement. The study was conducted following the Declaration of Helsinki and approved by the Tallinn Medical Research Ethics Committee of the Estonian National Institute for Health Development.

### 2.2. Study Protocol

The study included two measurements. Firstly, the participants came to the EEG laboratory of the Tallinn University of Technology where the EEG recordings were performed. Electroencephalography was recorded between about 6.30 and 9.00 am.

Secondly, on the same day, the participants visited the North Estonia Medical Centre to provide a blood sample. The blood samples were analyzed in the accredited laboratory of the North Estonia Medical Centre.

The participants were asked not to eat or drink coffee or tea in the morning before the study and to only drink water.

### 2.3. EEG Recordings

The resting-state eyes-closed EEG recordings were made in a shielded room. The room was dimly lit. The participants were in a relaxed eyes-closed supine position. The EEG signals were recorded for 6 min.

The Neuroscan Synamps2 acquisition system (Compumedics, Charlotte, NC, USA) was used for the EEG recordings. The EEG signals were recorded from 30 electrodes placed according to the extended international 10–20 system referenced to averaged mastoids. Electrode impedances were kept below 10 kΩ. Horizontal and vertical electrooculograms were used for monitoring eye movement. The raw EEG data were recorded in the 0.5–200 Hz frequency band at the sampling frequency of 1000 Hz.

### 2.4. EEG Processing

MATLAB (The MathWorks, Inc., Natick, MA, USA) software version R2023b was used for EEG data processing. First, the signal was re-referenced to Cz. Next, the raw EEG signal was filtered into the 2–47 Hz frequency band using a Butterworth filter with an attenuation of 100 dB in the stopband.

The traditional quantitative EEG markers theta (TBP), alpha (ABP), beta (BBP), and gamma (GBP) band powers are linear markers that describe the distribution of power in the EEG frequency spectrum. To calculate the traditional frequency band power markers, the signals were further filtered into delta (1–4 Hz), theta (4–8 Hz), alpha (8–12 Hz), beta (12–30 Hz), and gamma (30–48 Hz) frequency bands. Next, the signals were partitioned into 10 s segments. A qualified specialist inspected all segments, removing those containing ocular, muscular, or other artifacts. For each subject, the first 30 artifact-free segments (5 min in total) were selected for further analysis. Next, the average power for all EEG frequency bands was calculated for each subject and channel.

Higuchi’s fractal dimension is a nonlinear marker that describes the temporal dynamics of a signal and reflects the self-similarity of EEG signals. To reduce the computation time, the signal was down-sampled to 200 Hz. Segmenting and segment selection were performed identically as for the linear markers. Next, HFD was calculated in the time domain according to the original formulas [46]. From the initial time series, a new series *X*(1), *X*(2), *X*(3), …, *X*(*N*) was formed as follows:

(1) $$X_{k}^{m}:X\left(m\right),X\left(m+k\right),X\left(m+2k\right),\ldots ,X\left(m+int\left(\frac{N-m}{k}\right)\cdot k\right),\;\;\;\;\;\;m=1,\;2,\ldots ,\;k$$

The length *L_m_*(*k*) of the curve was calculated according to the formula:

(2) $$L_{m}\left(k\right)=\frac{1}{k}\left\lfloor \left(\sum_{i=1}^{\frac{N-m}{k}}\left[X\left(m+ik\right)-X(m+(i-1)\right]k\right)\frac{N-1}{\left(\frac{N-m}{k}\right)k}\right\rfloor$$

The length *L*(*k*) of the curve for the time interval *k* was defined as the average over the *k* values of *L_m_*(*k*), *m* = 1, 2, 3, …, *k*. If *L*(*k*) scales as *L*(*k*)~*k*^-FD^, the curve has the fractal dimension *FD*, which is calculated according to the following formula:

(3) $$FD=\frac{n\sum (x_{i}y_{i})-\sum x_{k}\sum y_{k}}{n\sum x_{k}^{2}-{\left(\sum x_{k}\right)}^{2}}$$

where *x_k_* = *ln*(1/*k*), *y_k_* = *ln L*(*k*), *k* = *k*_1_, …, *k_max_*, and *n* denotes the number of *k* values for which the linear regression is calculated (2 ≤ *n* ≤ *k_max_*). A scaling factor *k_max_* = 8 was used for the EEG analyses at the sampling frequency of 200 Hz [45,47]. The calculations were performed for each of the segments and averaged.

### 2.5. Statistics

The EEG markers were calculated for each participant in each EEG channel. Next, the correlation coefficients between the single-channel EEG markers and the blood biomarkers were calculated in each channel. The values of the correlation coefficients averaged over all channels were used for further statistical evaluation.

In healthy people, the ranges of the blood biomarkers’ variations are limited by the corresponding clinical reference limits. Therefore, alterations in the EEG markers related to the blood biomarkers are expected to be rather small, and a linear approach is appropriate. The Pearson correlation coefficient was used to calculate the correlations. The correlation between blood biomarkers and EEG markers can depend on the age and gender of the participants. The correlation coefficients corrected to partial correlation for age and gender were used in further statistical evaluation.

The significance of the correlation coefficients was assessed using Student’s t-test. The correlation of each blood biomarker with the five EEG markers was evaluated. Bonferroni correction for n = 5 multiple comparisons between a blood biomarker and the EEG markers determined the level of statistical significance *p* < 0.05/5 = 0.01.

## 3. Results

Table 1 presents the values of the blood biomarkers (glucose, protein, lipoprotein, HDL, LDL, C-reactive protein, and cystatin C) and EEG markers (TBP, ABP, BBP, GBP, and HFD) averaged over the group and the correlation coefficients of the markers with the age and gender of the participants.

The group average values of the blood biomarkers (except one) were within the reference limits, indicating the healthy state of the selected participants. The average level of LDL was somewhat higher. The standard deviations were high for the majority of the blood biomarkers as well as for the EEG markers. The correlation with age was weak for the majority of the blood biomarkers and showed an increase only for cystatin C. The correlation with gender showed a decrease only for HDL and an increase for lipoprotein in men.

**Figure 1:**
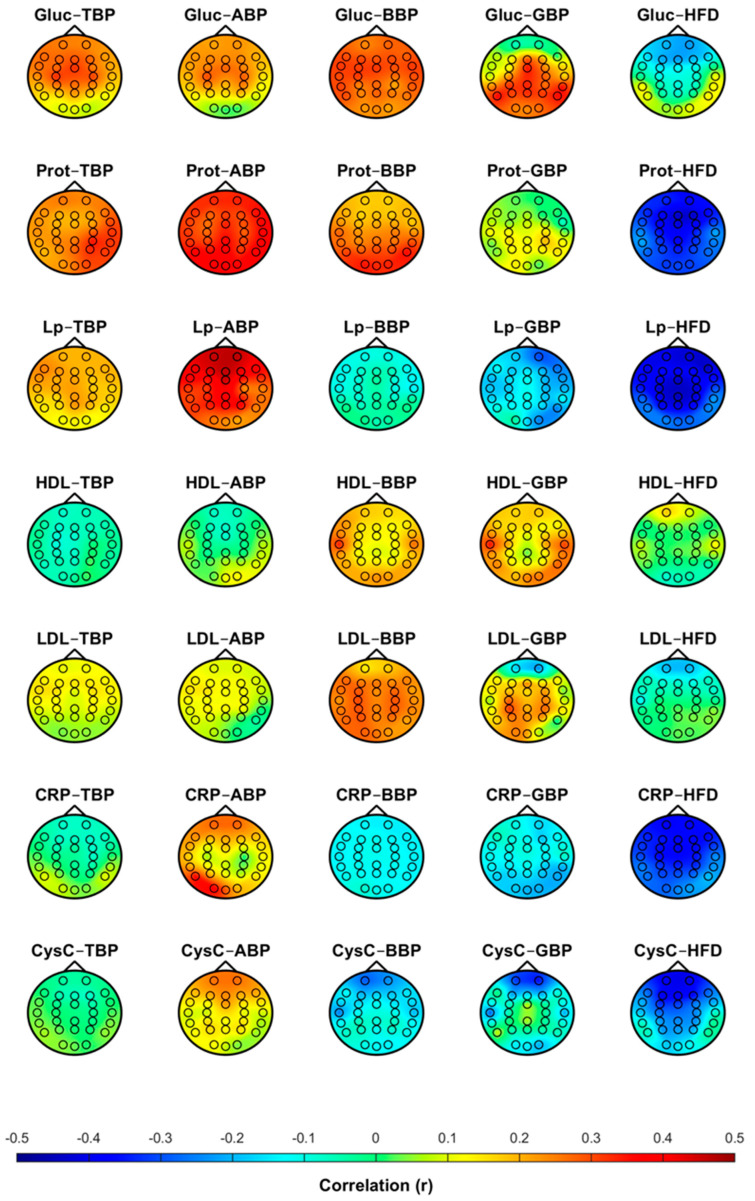
Distribution of the correlation coefficients over the brain (*n* = 52).

Figure 1 demonstrates the distribution of the level of correlation between the blood biomarkers and the EEG markers over the brain. The plotted correlation coefficients in different EEG channels showed that the correlation was similar over the whole brain. 

The level of correlation in the dominant brain areas varied for different biomarkers from the occipital region (glucose–GBP, protein–BBP) to the frontal region (glucose–ABP, glucose–BBP, glucose–HFD, protein–HFD, lipoprotein–HFD, CRP–HFD, and cystatin C–HFD). A positive correlation was found between the blood biomarkers and the EEG power markers, but the correlation was negative between the blood biomarkers and the EEG complexity marker HFD. The level of correlation was rather weak: the values of the correlation coefficients were lower than 0.4 in all cases (except for lipoprotein–HFD, whose correlation value reached 0.44).

**Figure 2:**
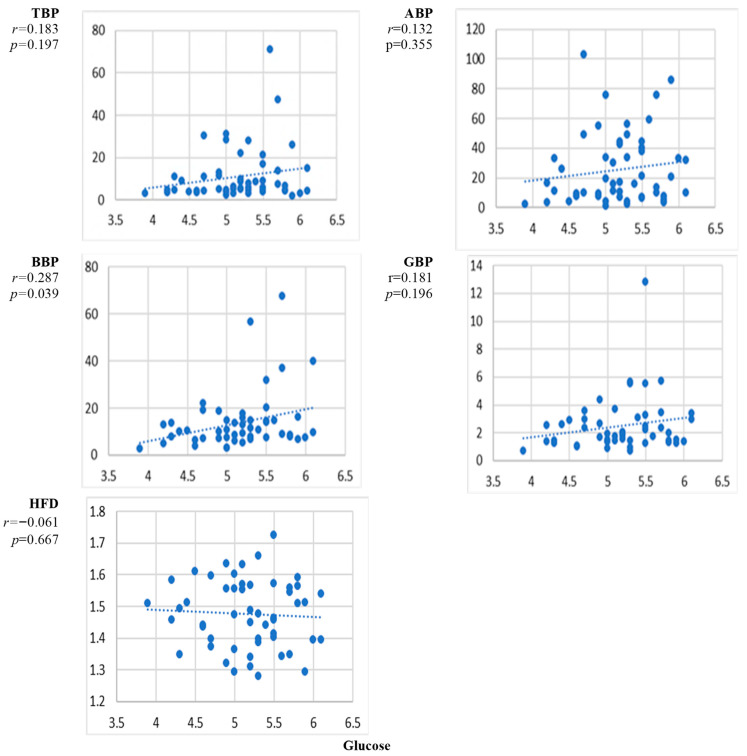
Scatter plots of the correlation between glucose concentration and EEG markers. *p* < 0.01 indicates statistical significance (*n* = 52).

Figure 2 presents the scatter plots of the correlation between the natural level of glucose and the EEG markers averaged over all channels and the corresponding correlation coefficients. The curves indicate some increase with the level of glucose for the EEG power markers and a minimal decrease for HFD, but the level of correlation did not reach the criteria of statistical significance *p* < 0.01 for any of the EEG markers.

**Figure 3:**
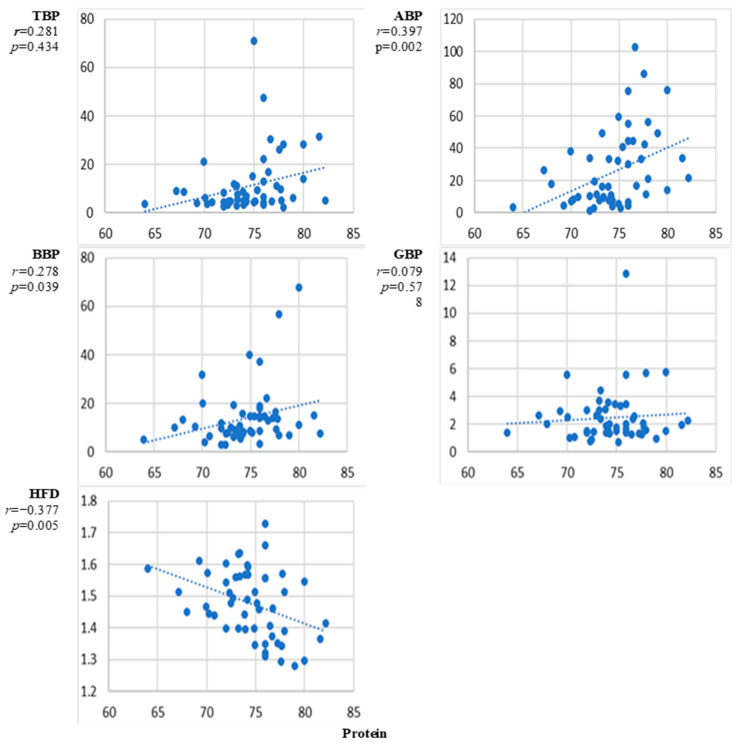
Scatter plots of the correlation between protein concentration and EEG markers. *p* < 0.01 indicates statistical significance (*n* = 52).

Figure 3 presents the scatter plots of the correlation between the natural level of protein and the EEG markers averaged over all channels and the corresponding correlation coefficients. The curves indicate an increase with the level of glucose for all EEG power markers. However, the correlation reached the level of significance *p* < 0.01 only for the alpha band power. A clear negative statistically significant correlation is evident between protein and HFD.

**Figure 4:**
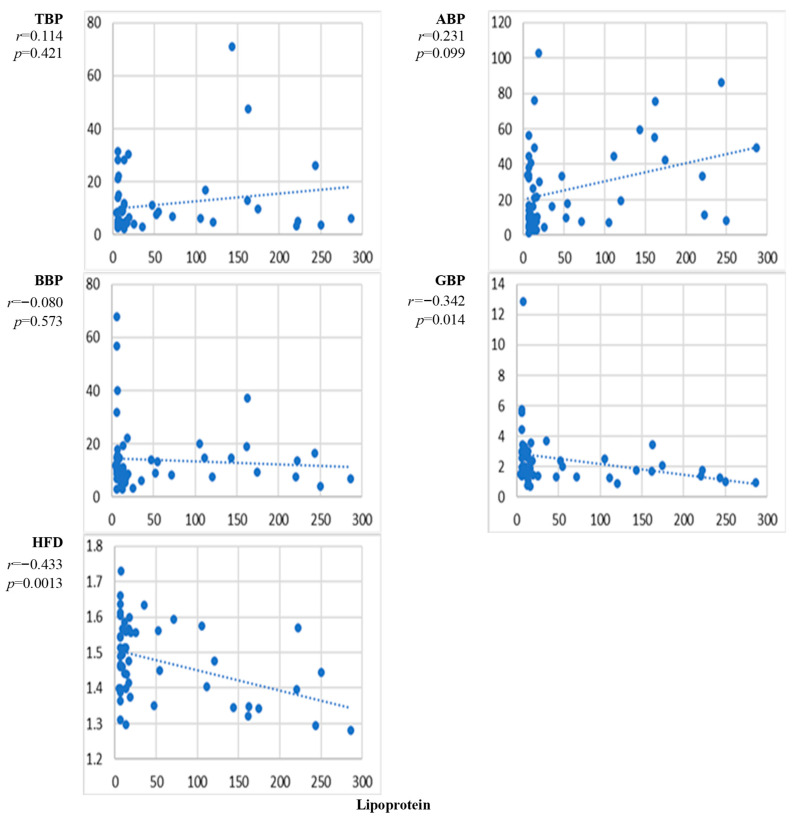
Scatter plots of the correlation between lipoprotein concentration and EEG markers. *p* < 0.01 indicates statistical significance (*n* = 52).

Figure 4 presents the scatter plots of the correlation between the natural level of lipoprotein and the EEG markers averaged over all channels and the corresponding correlation coefficients. Only minimal alterations with the level of lipoprotein can be noticed for TBP, BBP, and GBP. A somewhat higher but statistically insignificant increase is noticeable for the correlation between lipoprotein and ABP. The complexity of EEG reflected by HFD showed a clear and statistically significant decrease with the level of lipoprotein.

**Figure 5:**
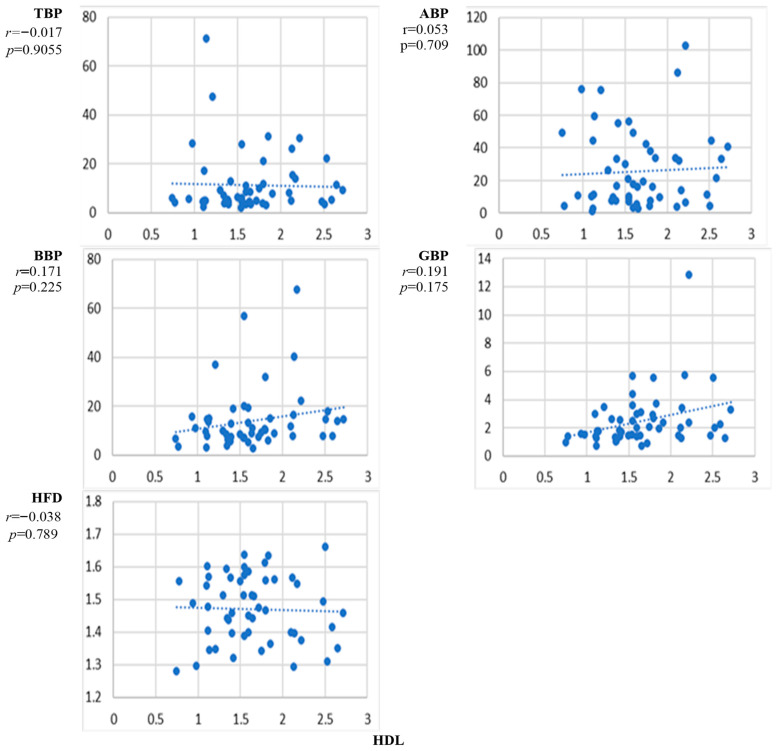
Scatter plots of the correlation between HDL concentration and EEG markers. *p* < 0.01 indicates statistical significance (*n* = 52).

Figure 5 presents the scatter plots of the correlation between the natural level of high-density lipoprotein HDL and the EEG markers averaged over all channels and the corresponding correlation coefficients. The correlation curves indicate an increase with the level of HDL for BBP and GBP. However, the correlation was statistically insignificant. No noticeable trends of alterations for TBP, ABP, and HFD were evident.

**Figure 6:**
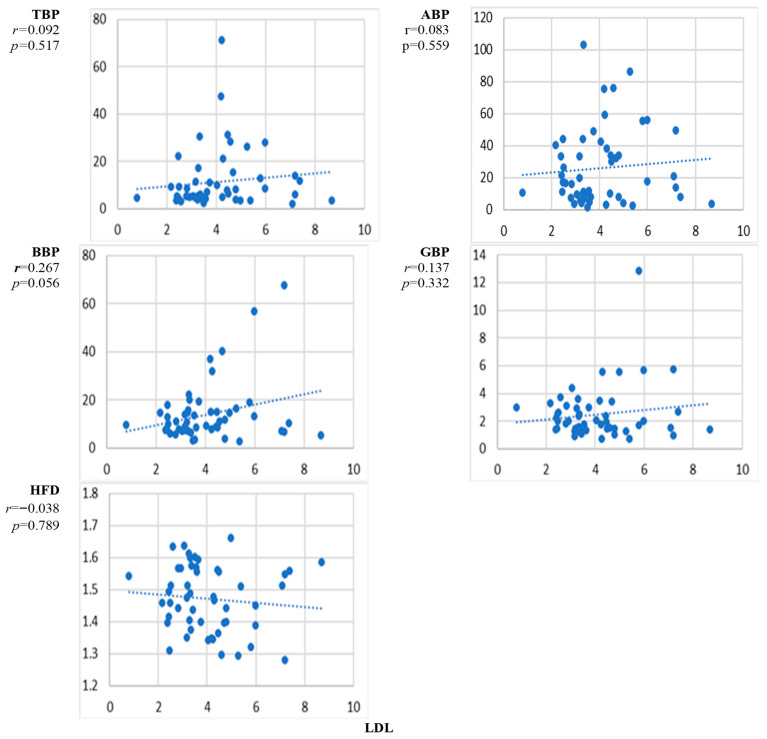
Scatter plots of the correlation between LDL concentration and EEG markers. *p* < 0.01 indicates statistical significance (*n* = 52).

Figure 6 presents the scatter plots of the correlation between the natural level of low-density lipoprotein LDL and the EEG markers averaged over all channels and the corresponding correlation coefficients. The increase with the level of LDL was most noticeable for BBP but did not reach the level of significance.

**Figure 7:**
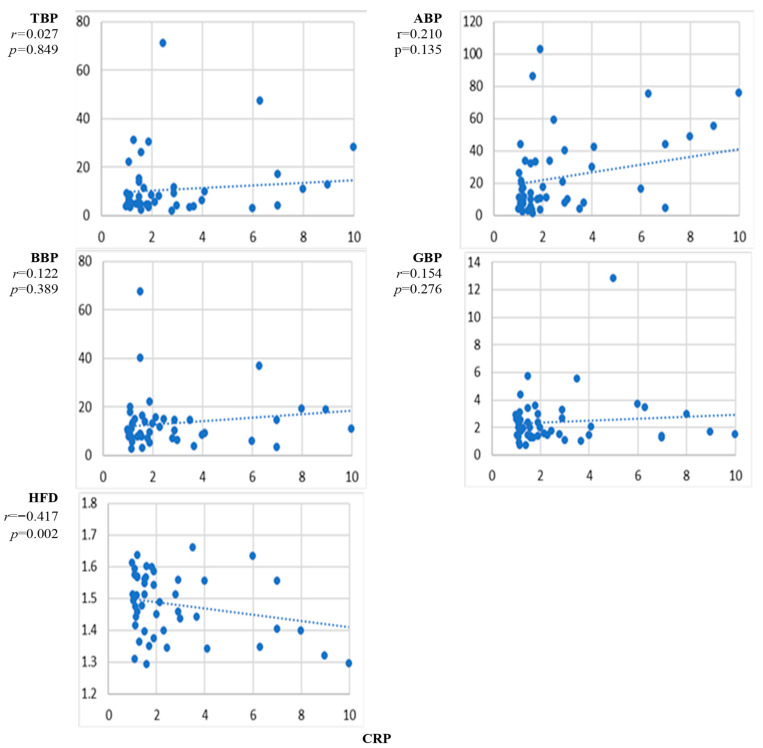
Scatter plots of the correlation between C-reactive protein concentration and EEG markers. *p* < 0.01 indicates statistical significance (*n* = 52).

Figure 7 presents the scatter plots of the correlation between the natural level of C-reactive protein and the EEG markers averaged over all channels and the corresponding correlation coefficients. Among the EEG power markers, ABP showed enhancement with the level of CRP, but the correlation was insignificant. The EEG complexity marker HFD decreased with the level of CRP, and the negative correlation between HFD and CRP was significant.

**Figure 8:**
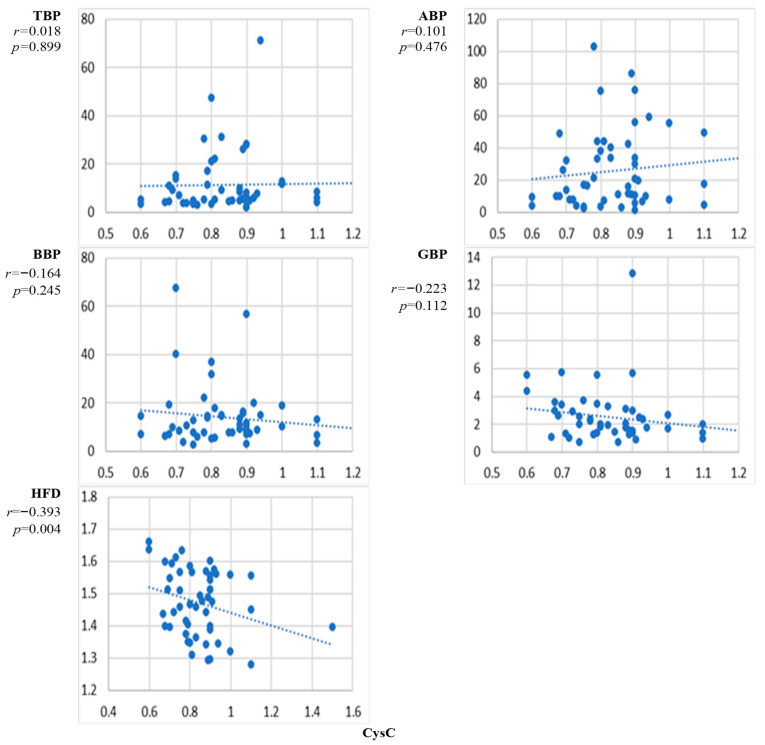
Scatter plots of the correlation between cystatin C concentration and EEG markers. *p* < 0.01 indicates statistical significance (*n* = 52).

Figure 8 presents the scatter plots of the correlation between the natural level of cystatin C and the EEG markers averaged over all channels and the corresponding correlation coefficients. The correlation between cystatin C and TBP, ABP, BBP, and GBP was not statistically significant. A decrease in HFD with the increasing level of cystatin C was evident, and the correlation was statistically significant.

The results presented in Figure 2, Figure 3, Figure 4, Figure 5, Figure 6, Figure 7 and Figure 8 show that the level of correlation met the criterion of statistical significance, *p* < 0.01, for four biomarkers, i.e., protein with ABP and HFD (Figure 3), lipoprotein with HFD (Figure 4), C-reactive protein with HFD (Figure 7), and cystatin C with HFD (Figure 8). The correlation between glucose, HDL, and LDL and any of the EEG markers did not reach the level of significance (Figure 2, Figure 5 and Figure 6). The correlation of ABP with protein was positive, but the correlation of HFD with protein, lipoprotein, CRP, and cystatin C was negative. The EEG power marker ABP showed correlation with only one blood biomarker, i.e., protein (Figure 3). The EEG complexity marker HFD showed correlation with four blood biomarkers: protein (Figure 3), lipoprotein (Figure 4), C-reactive protein (Figure 7), and cystatin C (Figure 8).

Figure 2, Figure 3, Figure 4, Figure 5, Figure 6, Figure 7 and Figure 8 show that the scattering was high for all linear as well as nonlinear EEG markers. The values of the EEG markers varied in wide intervals even with narrow intervals of the blood biomarkers. The character of the scattering was similar for the linear and nonlinear EEG markers.

## 4. Discussion

The results of the performed study demonstrate that the correlation between the natural level of blood biomarkers and EEG markers was similar in all EEG channels (Figure 1). The results show close correlation values in symmetric EEG channels of both hemispheres. These results are consistent with the results of previous studies performed on fasting and diabetic subjects, showing that alterations in biomarkers were evident in all EEG channels, with no interhemispheric differences [26,29]. The correlations in the dominant areas varied for different biomarkers and EEG markers (Figure 1). A higher positive correlation of ABP with protein was found in the occipital area, while with lipoprotein and glucose, it was found in the frontal area. A negative correlation of HFD with all biomarkers dominated in the frontal and temporal–parietal areas.

The data in Table 1 show an insignificant dependence for the majority of blood biomarkers and EEG markers on age, although the physiological parameters of people are known to change in aging. The weak correlation of the markers with age is due to a quite narrow age span of the middle-aged participants selected in the study (average age of 47.7 ± 11.4 years). Earlier studies on EEG fractality in healthy aging demonstrated that the dependence of HFD on age can be shaped as a parabola with a vertex around 50–60 years [48,49]. This knowledge explains the very weak correlation of the examined parameters with age in the current study. The correlation of the level of cystatin C with age is most probably related to the decrease in kidney function in aging.

The linear EEG markers (TBP, ABP, BBP) showed an increasing trend with the increasing level of glucose, protein, and lipoprotein (Figure 1). This result is consistent with previous studies performed on subjects with experimentally changed levels of blood biomarkers, where an increase in the EEG theta and alpha band powers at higher levels of glucose and protein was reported [26,28,31]. However, the correlation between the linear EEG markers and the blood biomarkers was statistically significant only between ABP and protein (Figure 3). In earlier studies, only an increase in EEG power was reported, but the correlation was not evaluated [26,28,31].

The level of correlation between the blood biomarkers and the EEG markers averaged over all channels reached the criteria of statistical significance, *p* < 0.01, for four blood biomarkers, i.e., protein with ABP and HFD (Figure 3), lipoprotein with HFD (Figure 4), C-reactive protein with HFD (Figure 7), and cystatin C with HFD (Figure 8). The correlation between glucose, HDL, and LDL with any of the EEG markers did not reach the level of significance (Figure 2, Figure 5 and Figure 6).

The linear EEG power markers reveal a correlation only with one blood biomarker, ABP with protein (Figure 3). The EEG complexity marker, HFD, is correlated with four blood biomarkers: protein (Figure 3), lipoprotein (Figure 4), C-reactive protein (Figure 7), and cystatin C (Figure 8).

The correlation of the linear EEG marker ABP with protein was positive, while the correlation of the nonlinear EEG marker HFD with protein, lipoprotein, CRP, and cystatin C was negative. The change in the character of the correlation indicated by the EEG power and the EEG complexity described by HFD was demonstrated also in our previous studies, where a negative correlation between HFD and EEG power markers was reported [45]. A negative correlation was also shown between EEG complexity markers and psychological test scores [50]. No published data are available about the relationship between EEG complexity and blood biomarkers.

The negative correlation of HFD with protein, lipoprotein, cystatin C, and C-reactive protein provides a new insight into the ability of EEG signals to reflect brain functioning. The effect of protein on brain functioning can be related to its role in brain plasticity [51]. Protein negatively regulates synapse formation and plasticity [52], which may be reflected in a reduction of EEG complexity. An elevated concentration of lipoproteins leads to degeneration in the brain, cognitive decline, and decreased complexity [34,53]. A decrease in EEG complexity with the increase in C-reactive protein would be expected due to the worsening of liver function that can affect the brain [54,55].

C-reactive protein is implicated in cognitive impairment, Alzheimer’s disease, and depression [54,55,56]. The complexity of the brain is expected to decrease with cognitive impairment [57,58]. This knowledge supports the finding that the correlation between HFD and C-reactive protein was negative. The level of cystatin C is regulated by the kidneys, and the worsening of kidney function can lead to cognitive, psychiatric, and motor impairments [41,42,43,59]. Cystatin C level is associated with the future risk of cardiovascular disease and mortality [60]. A high level of cystatin C indicates greater disease severity, faster progression rate, and shorter survival in ALS patients [61]. A low kidney functioning, indicated by an increase in cystatin C level, results in decreased brain complexity, reflected by the negative correlation between cystatin C and HFD.

The high level of variation in the individual responses of the EEG markers to the level of blood biomarkers (Figure 2, Figure 3, Figure 4, Figure 5, Figure 6, Figure 7 and Figure 8) can be related to the individual sensitivity of brain functioning to the level of different blood biomarkers. The conclusion that the threshold of biomarkers causing changes in brain physiology and EEG varies between individuals has been derived also by other authors [2,9]. The variability of the sensitivity of brain functioning to blood biomarkers contributes to the interindividual variation of the responses of EEG markers.

Various EEG markers demonstrated different sensitivity to different blood biomarkers. This can be explained by the association of different blood biomarkers with different processes in the brain. ABP was correlated with one blood biomarker, protein. HFD revealed a correlation with four blood biomarkers, i.e., protein, lipoprotein, C-reactive protein, and cystatin C. HFD was the only EEG marker that revealed the effect of liver and kidney biomarkers on brain functioning.

The performed study has some limitations. The first limitation is the high variability of the measured data (Figure 2, Figure 3, Figure 4, Figure 5, Figure 6, Figure 7 and Figure 8). The high variability of the data is most probably related to interindividual differences. The sensitivity of brain functioning to alterations in the concentration of blood biomarkers has been reported to differ between individuals [22,29]. Therefore, identical levels of blood biomarkers can be related to different strengths of the responses in the brain.

The second limitation is the small range of the values of the concentrations of blood biomarkers in healthy people. The range of these concentrations in healthy people is limited by the clinical reference levels. The small range of the values of blood biomarkers decreases the statistical strength of the correlation and the possibility of revealing their correlation with EEG markers.

The effects of the first and second limitations can be reduced by performing studies on larger groups of healthy people. The correlation between blood biomarkers and the state of the brain in healthy people needs further investigation in larger groups of participants and more detailed studies using different brain markers.

## 5. Conclusions

The results of the current study show that the EEG markers are affected by the natural level of various blood biomarkers in healthy people. The analysis of the results led to the following conclusions:

1. The complexity of the EEG signals indicated by the nonlinear dynamics marker HFD is more sensitive to the natural level of blood biomarkers than the EEG power. Only one of the four linear EEG power markers was correlated with one biomarker (ABP with protein). One nonlinear EEG marker, HFD, was correlated with four blood biomarkers (protein, lipoprotein, C-reactive protein, and cystatin C).
2. The nature of the relationship between blood biomarkers and EEG signals differs in signal power and complexity. The level of EEG power, indicated by ABP, increased with a higher level of protein. The complexity of the brain reflected by HFD decreased with enhanced levels of protein, lipoprotein, C-reactive protein, and cystatin C.
3. The impact of the natural level of blood biomarkers on EEG in healthy people, despite being statistically significant for some blood biomarkers, is rather weak. The correlation coefficients between the EEG markers and the blood biomarkers were lower than 0.4. The impact on EEG increased with the imbalance in blood chemicals.

The reported findings suggest that the state of the brain of healthy people can be affected by the natural level of their blood biomarkers related to their everyday dietary habits. This novel knowledge may be important in providing information about people quality of life and well-being.

## Figures and Tables

**Table 1:** Average values, standard deviations, relative standard deviations, and correlation coefficients with age *r*_a_ and gender *r*_g_ of the measured blood biomarkers and calculated EEG markers for the group (*n* = 52).

Markers, Units, Reference Values		Average	StDev	Rel StDev %	*r*_a_	*r*_g_
**Gluc** mmol/L	4.1–6.0	5.18	0.53	10.2	0.052	−0.004
**Prot** g/L	64–87	74.53	3.51	4.7	−0.136	0.172
**Lp** nmol/L	<75	55.12	77.6	140.9	−0.102	0.384
**HDL** mmol/L	>1.2	1.66	0.49	29.7	0.002	−0.518
**LDL** mmol/L	<3	4.10	1.57	38.3	0.035	−0.006
**CRP** mg/L	<5	1.79	2.94	164.8	0.143	0.160
**CysC** mg/L	0.47–1.09	0.84	0.15	17.4	0.304	0.145
**TBP**		112.3	126.2	112.3	−0.212	0.136
**ABP**		256.8	239.0	93.07	−0.058	0.189
**BBP**		139.5	123.6	88.5	0.014	−0.124
**GBP**		24.8	19.4	78.2	0.013	−0.118
**HFD**		1.47	0.11	7.48	−0.040	−0.061

## Data Availability

Data are available upon request from the corresponding author.
